# Recovery-focused care planning and coordination in England and Wales: a cross-national mixed methods comparative case study

**DOI:** 10.1186/s12888-016-0858-x

**Published:** 2016-05-16

**Authors:** Alan Simpson, Ben Hannigan, Michael Coffey, Sally Barlow, Rachel Cohen, Aled Jones, Jitka Všetečková, Alison Faulkner, Alexandra Thornton, Martin Cartwright

**Affiliations:** Centre for Mental Health Research, School of Health Sciences, City University London, Northampton Square, London, EC1V 0HB UK; East London NHS Foundation Trust, 9 Alie St, London, E1 8DE UK; School of Healthcare Sciences, Cardiff University, Cardiff, CF10 3XQ UK; Department of Public Health Policy and Social Sciences, Swansea University, Singleton Park, Swansea, SA2 8PP UK; Faculty of Health and Social Care, The Open University, Walton Hall, Milton Keynes, Buckinghamshire, MK7 6AA UK; Independent Service User Researcher Consultant, London, UK; Centre for Health Services Research, School of Health Sciences, City University London, Northampton Square, London, EC1V 0HB UK

**Keywords:** Care coordination, Care planning, Case study, Community mental health, Recovery, Personalisation, Therapeutic relationships, UK

## Abstract

**Background:**

In the UK, concerns about safety and fragmented community mental health care led to the development of the care programme approach in England and care and treatment planning in Wales. These systems require service users to have a care coordinator, written care plan and regular reviews of their care. Processes are required to be collaborative, recovery-focused and personalised but have rarely been researched. We aimed to obtain the views and experiences of stakeholders involved in community mental health care and identify factors that facilitate or act as barriers to personalised, collaborative, recovery-focused care.

**Methods:**

We conducted a cross-national comparative study employing a concurrent transformative mixed-methods approach with embedded case studies across six service provider sites in England and Wales. The study included a survey of views on recovery, empowerment and therapeutic relationships in service users (*n* = 448) and recovery in care coordinators (*n* = 201); embedded case studies involving interviews with service providers, service users and carers (*n* = 117) and a review of care plans (*n* = 33). Quantitative and qualitative data were analysed within and across sites using inferential statistics, correlations and framework method.

**Results:**

Significant differences were found across sites for scores on therapeutic relationships. Variation within sites and participant groups was reported in experiences of care planning and understandings of recovery and personalisation. Care plans were described as administratively burdensome and were rarely consulted. Carers reported varying levels of involvement. Risk assessments were central to clinical concerns but were rarely discussed with service users. Service users valued therapeutic relationships with care coordinators and others, and saw these as central to recovery.

**Conclusions:**

Administrative elements of care coordination reduce opportunities for recovery-focused and personalised work. There were few common understandings of recovery which may limit shared goals. Conversations on risk appeared to be neglected and assessments kept from service users. A reluctance to engage in dialogue about risk management may work against opportunities for positive risk-taking as part of recovery-focused work. Research to investigate innovative approaches to maximise staff contact time with service users and carers, shared decision-making in risk assessments, and training designed to enable personalised, recovery-focused care coordination is indicated.

**Electronic supplementary material:**

The online version of this article (doi:10.1186/s12888-016-0858-x) contains supplementary material, which is available to authorized users.

## Background

### Policy context

In England and Wales the organisation and delivery of health and social care is changing and diverging. In England, greater use is being made of the market and of private care providers, and complex arrangements are now in place for quality and accountability [1]. In Wales, where authority is devolved, most health provision continues to be provided by public bodies who are not expected to act in competition [2]. In both countries, policy for mental health has become a relative priority and service development has been rapid [3]. Whilst clear differences in emphasis in England and Wales are found, shared approaches to values-based practice also exist. These include commitments to care which is responsive to the individual, and to services which are well-planned and coordinated.

Formal arrangements for care planning and coordination have existed for a quarter of a century. In England, the care programme approach (CPA) was introduced in 1991 as a type of case management [4] and has since been revised and refocused [5]. The CPA came later to Wales [6] but has now been replaced by a legislative framework which places care and treatment plans (CTPs) and care coordination on a statutory footing [7], in contrast to the non-statutory guidance in England. Systems in both countries require practitioners to comprehensively assess health and social care needs and risk, to plan and coordinate care, and to regularly monitor and review. Current policy in both countries also emphasises that these processes should be carried out in ways which promote recovery [8], and which reflect commitments to collaborative, and individually tailored or personalised care [5, 9].

### Evidence base

Systematic reviews of case management in community mental health exist (e.g., [10]), but research has not focused on the relationships between care planning and coordination and recovery outcomes. Findings from early studies of the CPA in England pointed to excessive bureaucracy, contributing to limited opportunities for staff to provide therapeutic interventions [11, 12]. Large-scale audits suggest that care planning and coordination are subject to significant local variation and limited involvement of service users and carers^1^ [13, 14].

This relative lack of involvement of service users and carers in the care planning and coordination process is important. Collaborative, therapeutic relationships are associated with positive service user outcomes in mental health care [15, 16]. Partnership and strong working alliances between case managers and empowered people with long-term mental health difficulties help reduce symptoms, improve functioning and social skills, promote quality of life, enhance medication compliance and raise satisfaction levels [17]. Care negotiated within a trusting relationship is key [18] and may influence users’ perceptions of stigma [19].

## Aim

Against this policy and evidence background the cross-national, multi-site, comparative study drawn on in this paper aimed to identify and describe factors associated with collaborative, personalised, recovery-focused care planning and coordination in community mental health services. The key objectives addressed in this paper are to:

1. Investigate service users’, informal carers’, practitioners’ and managers’ views and experiences of care planning and coordination.
2. Measure service user and staff perceptions of recovery-oriented practices.
3. Measure service users’ views of the quality of therapeutic relationships and perceptions of empowerment.
4. Compare experiences between sites and between the two countries to explore organisational and national policy-level influences.

It was hypothesised that ratings of recovery-oriented practices, therapeutic relationships and empowerment would be higher in those organisations where participants described care planning processes that were more collaborative, personalised and recovery-focused.

## Theory, design and sampling

**Fig. 1:**
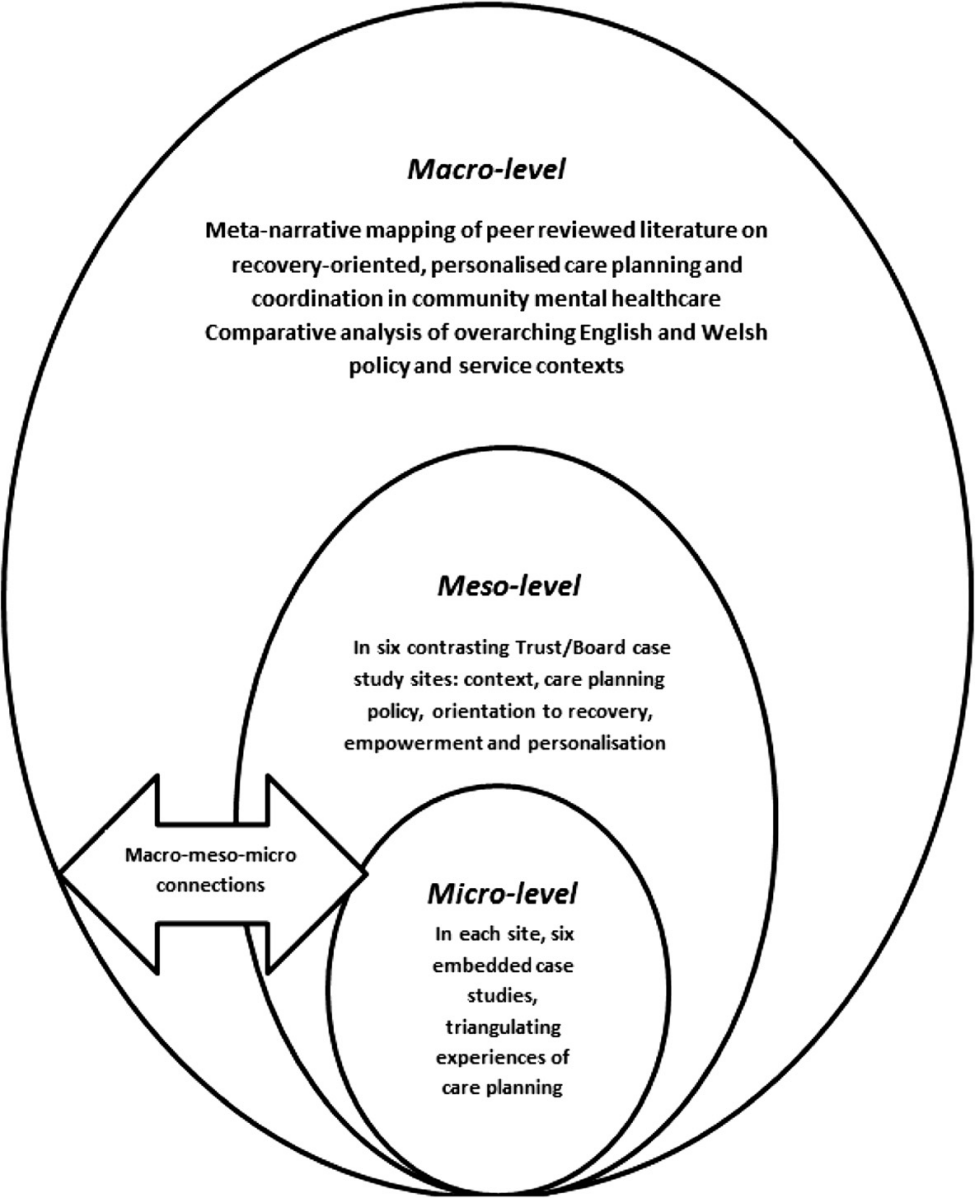
Diagram of study design with embedded case studies

A full protocol for the study [20], and a full report with detailed appendices have both been published [21]. In summary, informed by systems ideas which emphasise connections between macro, meso and micro levels of organisation [22] a cross-national comparative study employing a concurrent transformative mixed-methods approach with embedded case studies was used [23–25] (see Fig. 1). In-depth micro-level case studies of everyday ‘frontline’ practice and experience with detailed qualitative data from interviews and reviews of individual care plans are nested within larger meso-level survey data sets, senior-level interviews and policy reviews in order to provide potential explanations and understanding. The macro-level was considered to be the contrasting national policy and organisational contexts found in England and Wales that served to shape local provision.

Access was secured to six National Health Service (NHS) organisations providing mental health care: four Trusts in England (pseudonymously referred to as ‘Artois’, ‘Dauphine’, ‘Languedoc’ and ‘Provence’) and two Local Health Boards (LHBs) in Wales (‘Burgundy’ and ‘Champagne’). Sites were selected to reflect geographical, population and urban/rural diversity (see Table 1). With help from local collaborators and research support staff, access was secured to 20 community mental health teams (CMHTs) across the six sites, with one specific team within each of these sites identified for in-depth case studies. In the UK CMHTs are the main vehicles for the local provision of secondary mental health care, and are typically funded by NHS and local authority organisations and staffed by health and social care workers, including psychiatrists, mental health nurses, occupational therapists, psychologists and social workers. Sampling criteria for CMHTs included the routine provision of care to adults, having a team manager in post and not being scheduled for merger or closure.

**Table 1:** Summary of site characteristics and data collection across the six meso-level case study sites

Site (country)	Characteristics of the Site		Questionnaire Returns		Interviews				
			Care Coordinators	Service Users	Senior Managers	Senior Practitioners	Care Coordinators	Service Users ^a^	Carers
Artois (England)	Covers a large and predominantly rural area, serving a population of around 1.6 million. Adult psychiatric admissions are provided in 7 hospitals and 6 rehabilitation units, and 13 adult services CMHTs^b^.		38	70	2	4	2	4	0
Burgundy (Wales)	Covers a wide geographical area with a mix of urban and rural communities, serving a population of around 500,000. Care is provided through 3 psychiatric hospitals, 1 community rehabilitation unit, 8 adult services CMHTs and a range of specialist services.		37	75	2	3	3	5	4
Champagne (Wales)	Covers two contrasting areas: one urban and fairly ethnically diverse, the other rural and predominantly White British. Serves approximately 500,000 people through 2 psychiatric hospitals and 8 adult services CMHTs.		31	72	1	5	5	6	4
Dauphine (England)	Covers an extremely densely populated and multicultural urban area. Serves approximately 750,000 people through 3 psychiatric hospitals and 10 adult services CMHTs.		33	61	2	5	6	6	2
Languedoc (England)	Covers a largely rural area, serving a population of around 735,000 people. Provides adult services through 7 CMHTs and 2 psychiatric hospitals.		28	92	3	5	6	6	2
Provence (England)	Covers a predominantly rural area, serving a population of around 1.5 million. Adult inpatient services are provided from 6 hospital sites, and community services through approximately 30 CMHTs.		34	78	2	5	6	6	5
		Totals	201	448	12	27	28	33	17

Key: ^a^Service user interviews included a narrative review of their individual written care plan, conducted with the service user. ^b^ *CMHTs* Community Mental Health Teams

## Methods

### Macro-level data

To compare and contrast the national, macro-level, policy contexts across the two countries, government websites were systematically searched for current and past policy and guidance documents.

### Meso-level data

In each of the six, meso-level, case study sites local documents (e.g., organisational policies) were accessed and treated as contextual data. Semi-structured, audio-recorded interviews were conducted with up to seven purposively sampled senior managers and practitioners, designed to include a range of professional disciplines including consultant psychiatrists, senior mental health nurses, psychologists, social workers and occupational therapists. The interview schedule was devised in consultation with members of the project advisory group (PAG) and lived experience advisory group (LEAG) (made up of service users and carers with personal experience of care planning and coordination), and informed by the policy and literature reviews. Schedules contained 15 main questions (with prompts) reflecting the aims of the study; topics included care planning and care coordination processes, orientations to recovery, safety and risk, and personalised care (see Additional files: 1, 2, 3 and 4). Minor amendments to the schedule were made following piloting.

**Fig. 2:**
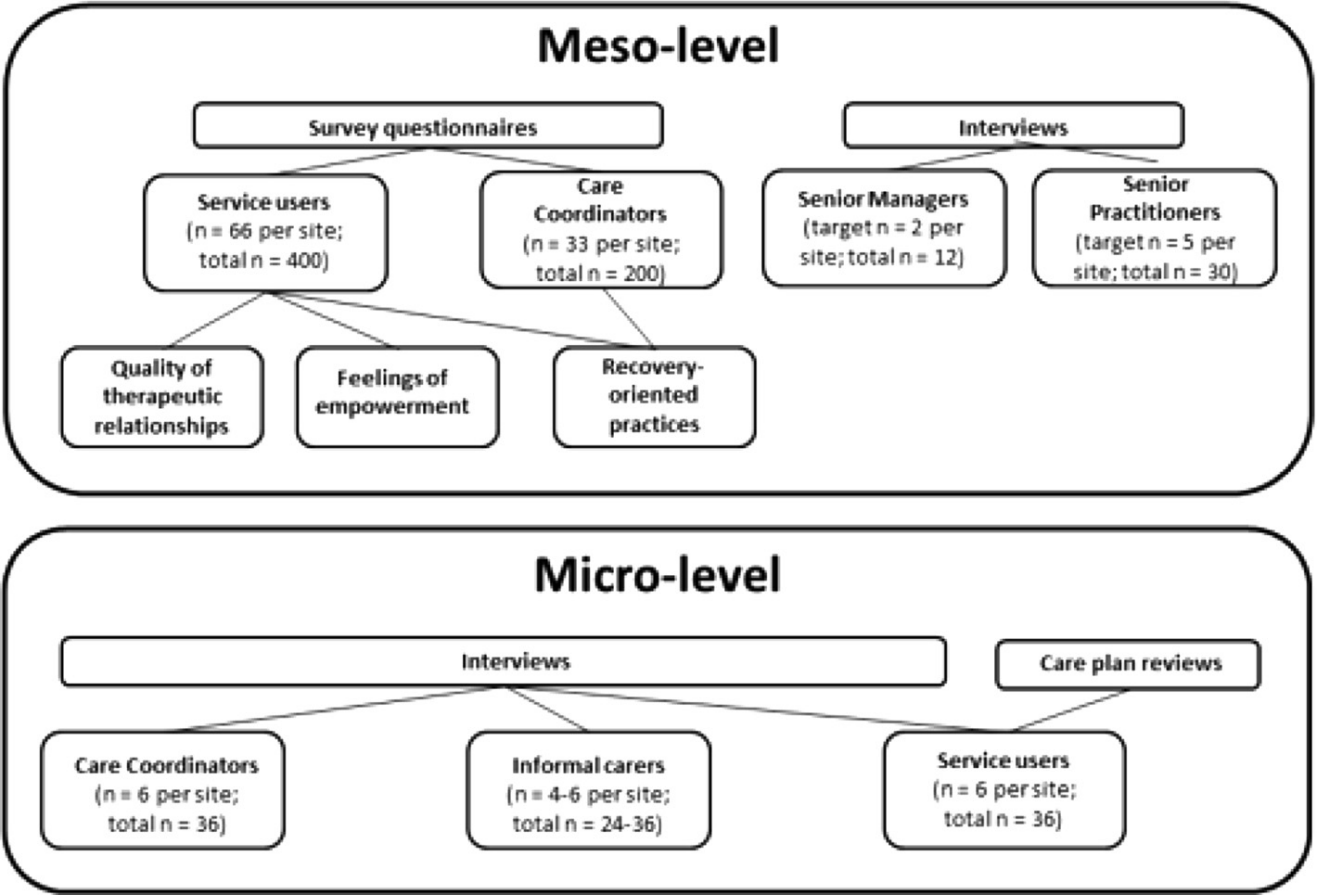
Meso- and micro-level data collection targets

Quantitative data relating to recovery, therapeutic relationships and empowerment were generated using survey methods from a total sample of 448 service users and 201 care coordinators, in line with the reported power calculation [20]. Measures used are outlined below. Sample targets and response rates by site are shown in Fig. 2 and Table 1 respectively.

The internal consistency of the scales and sub-scales used as outcome measures was assessed using Cronbach’s alpha for complete cases (i.e. those with complete data for a particular scale or sub-scale prior to mean replacement of missing values). In line with accepted practice an alpha of 0.7 or above was considered acceptable for the purpose of scale construction [26].

- The Recovery Self-Assessment Scale (RSA) [27]: a 36-item self-administered questionnaire measuring the extent of recovery-oriented practices, completed by service users and care coordinators (case managers in UK). Cronbach’s alpha for the Total RSA score, for service users was 0.97 (*N* = 139) and care coordinators was 0.95 (*N* = 156); Life Goals subscale, for service users was 0.93 (*N* = 253) and care coordinators 0.88 (*N* = 187); Involvement subscale, for service users 0.91 (*N* = 231) and care coordinators 0.84 (*N* = 175); Diversity of Treatment options subscale, for service users 0.83 (*N* = 251) and for care coordinators 0.75 (*N* = 187); Choice subscale, for service users 0.78 (N-352) and for care coordinators 0.67 (N = 186) and Individually tailored services subscale, for service users 0.87 (*N* = 216) and for care coordinators 0.72 (*N* = 188).
- The Scale To Assess the Therapeutic Relationship (STAR-P) [28]: a 12-item self-administered questionnaire assessing therapeutic relationships in community mental health, completed by service users. Cronbach’s alpha for the total STAR-P for service users was 0.91 (*N* = 391); Positive Collaboration subscale, 0.93 (*N* = 414); Positive clinician input subscale, 0.72 (*N* = 413) and Non-Supportive clinician input subscale, 0.63 (*N* = 411).
- The Empowerment Scale (ES) [29]: a 28-item self-administered questionnaire with five subscales measuring self-esteem, power, community activism, optimism and righteous anger, completed by service users. Cronbach’s alpha for the total empowerment score for service users was 0.84 (*N* = 371); Self-esteem-self-efficacy subscale, 0.92 (*N* = 407); Power-Powerlessness subscale 0.60 (*N* = 401); Community activism and autonomy subscale, 0.64 (*N* = 406); Optimism and control, 0.63 (*N* = 413) and Righteous anger, 0.45 (*N* = 418).

Questionnaires and a demographic sheet were distributed in the post to service users and distributed to staff in clinical meetings in each of the six main participating CMHTs, and to staff and service users in other CMHTs in each service provider site as necessary until target numbers of returns had been reached. In total, 20 CMHTs took part across the six sites. Survey packs for service users, each containing a copy of the three measures and participant information sheet, were mailed directly to home addresses and contained a paid-for, return-addressed envelope. Reminder letters were mailed out to all service users within three weeks.

### Micro-level data

Data at the micro-level, where face-to-face care is provided and received, were generated through audio-recorded semi-structured interviews conducted with each of the service users randomly sampled and recruited from the single target CMHT in each of the six case study sites. Clinical Studies Officers and Research Nurses supported the randomisation process by obtaining a full list of service users under the care of each CMHT (subject to CPA/CTP) grouped by care coordinator. Up to four service users per care coordinator were randomly selected from the sampling list using the RAND function in Microsoft Excel. Once a service user had agreed to participate, they were asked to identify an informal carer who might be approached for interview. Additionally, with participating service users’ permission, written care plans were accessed, and anonymised data extracted using an agreed template developed in consultation with the PAG and LEAG and drawing on relevant literature [30]. Sample targets and response rates by site are shown in Fig. 2 and Table 1 respectively.

## Ethics and approvals

The study received a favourable NHS Research Ethics opinion from the National Research Ethics Service (NRES) Yorkshire and The Humber Committee – Sheffield (Ref: 13/YH/0056A). Research governance approvals were also secured from each of the six participating NHS care provider organisations.

Care was taken throughout to protect participants’ and researchers’ wellbeing and to protect the identities of individuals and organisations. Lists of service users under the care of the selected CMHT and subject to the CPA/CTP, were checked with the responsible psychiatrist or team leader to prevent inappropriate mailings (e.g., if someone had recently died or been admitted to hospital). Questionnaires and interview invitations to service users were sent on the project team’s behalf by local research support staff authorised to access patient identifiable data. Participant information sheets and summaries of the study in multiple languages accompanied survey packs, and made clear individuals’ rights not to participate, how to withdraw, and arrangements for the preservation of anonymity and confidentiality. Information sheets accompanied all interview invitations and all interviewees signed consent forms. The study management team ensured quality control in the organisation and conduct of interviews, such as complying with lone worker policies and supporting researchers in receiving the appropriate training, supervision and opportunities for debriefing and structured reflection.

## Public and patient involvement

The study design and delivery benefited from the full involvement of co-investigator and independent service user researcher Alison Faulkner and from consultation with 14 members of the Service User and Carer Group Advising on Research (SUGAR) based at City University London [31]. The project LEAG was facilitated by Alison Faulkner and consisted of 10 service users and one carer, drawn from across both England and Wales. It met throughout the life of the project and advised on all aspects of it, including submissions to the research ethics committee, interview schedules, recruitment strategies, service user researcher experiences and initial analysis. One service user and the carer member also represented the LEAG on the PAG, along with Alison Faulkner. Five people with lived experience of mental ill-health were employed to work on the study with a primary role to help recruit and conduct interviews with user and carer participants.

## Data management and analysis

Reflecting the systems thinking underpinning the project, analysis was informed by the intention to investigate the macro-, meso- and the micro-levels of data and the connections, comparisons and contrasts between them. Qualitative and quantitative data in each of the six provider sites were considered on a within-group basis prior to a cross-case analysis aimed at identifying common themes and divergences. The between-group analysis of the quantitative data compared service users and care coordinators across sites on key markers of the service user experience (recovery-oriented care, therapeutic relationship and empowerment). The quantitative analyses were conducted alongside the qualitative analyses in a convergent parallel design that facilitates the integration of mixed methods data [23]. The large scale survey data paints with broad brush strokes while the interview data offers a more fine-grained rendering. This is a pragmatic approach to mixed method research that can generate a more complete understand of complex phenomena or processes. Quantitative and qualitative data analyses were conducted independently and subsequently synthesised to create a more complete understanding of the links across micro, meso and macro levels than either approach could achieve alone.

Questionnaire data from all sites were entered into SPSS version 21 [32], and were checked and cleaned by a second researcher prior to analysis. Distributions of the scale outcomes were assessed for normality using descriptive quantitative measures of skewness and kurtosis. We assessed normality for 16 outcomes for service users and six for staff, pooled across sites. Overall, this showed that the outcomes for both groups approximated the normal distribution. Deviations from normality were few in number (3 of 22 scale outcomes exceeded the conservative criteria of +/− 1) and small in the extent of deviation (all scales were within +/− 2). Parametric tests are robust to minor deviations from normality and were deemed appropriate in light of the skew and kurtosis values observed [33].

A missing value analysis for the 22 scale outcomes identified moderate to high levels of missing data, not missing at random, on a small proportion of items (mean level of missing data across the 22 scales/subscales was 19.9 %, range from 6.5 to 69 %). The service user version of the RSA questionnaire, in particular, had a relatively large degree of missing data. We used mean replacement to avoid unnecessary loss of cases from the analysis (i.e. missing values on a scale were replaced with the mean of the available items for that scale and that participant). There is no consensus in the literature on what level of missing values can be replaced in this way without over-fitting the model therefore we conducted a series of sensitivity analyses for each scale to determine the effect of using mean replacement in the primary analyses at different levels or replacement ranging from 20 % (i.e. the equivalent of replacing two missing values on a 10 item scale) to 50 % (i.e. the equivalent of replace 5 missing values on a 10 item scale). We compared the impact of these different levels of mean replacement to a complete case analysis using only participants with no missing values on a particular scale or sub-scale. This assessment of the impact of mean replacement on the calculated p-values and associated effect sizes suggested that mean replacement of up to 50 % did not produce any substantive differences compared to the either complete case analysis or a lower level of mean replacement. Allowing up to 50 % mean replacement on a single outcome maximised the number of cases included in the analyses (i.e. reduced loss of cases) with minimal impact on the key statistical parameters and the inferences drawn.

Descriptive statistics were calculated for the three questionnaires, and where appropriate scores were compared against reference values (STAR-P and ES). Unadjusted one-way analyses of variance (ANOVA) were conducted to compare differences between the six sites on the RSA, STAR-P and ES measures. A series of one way analyses of covariance (ANCOVA) were then completed to adjust for potential confounders. Two different classes of co-variates were selected for each participant group, demographics and service-related measures. Demographic co-variates for service users were age, gender, ethnicity, and relationship status, and the service-related co-variate was time in mental health services. Demographic co-variates for staff were age, gender and ethnicity, and the service-related co-variates were time working in mental health services and time as a care coordinator. These variables were selected in light of variation in the population characteristics between the six sites and their potential impact on the reported outcome measures. Where ANOVAs or ANCOVAs suggested between-group differences, Tukey post hoc tests were used to examine where the differences lie.

Correlations were carried out to identify if there was a relationship between outcome measures and to determine relationships amongst service users on recovery-oriented focus, empowerment and the quality of therapeutic relationships. Pearson’s correlations were used to assess the associations between the RSA and STAR-P, RSA and ES and STAR-P and ES for all participants and by individual site. These were interpreted in line with Cohen’s criteria [34] for small (*r* = 0.10), medium (*r* = 0.30) and large (*r* = 0.50) effect size. For all analyses statistical significance was set at a level of 0.05.

All digital interview recordings were professionally transcribed and any identifying information redacted, before being imported into QSR International’s NVivo 10 qualitative data analysis software [35] for analysis using the framework method [36, 37]. Multiple transcripts were read by members of the research team to aid familiarisation, with the framework matrix developed *a priori* from the interview schedules. Sections focused on organisational background and developments, care planning, recovery, personalisation, and recommendations for improvement. Each matrix section also had an ‘other’ column for the inclusion of data-led emergent categories. Piloting of summarising and charting processes was followed by a careful charting of all transcripts with cross-checking involving multiple team members. Second-level summarising allowed the further refining of data and the identification of commonalities and differences within and between sites and groups of participants. Summarised data from each of the embedded micro-level service user/carer/care coordinator case studies in each site were compared against each care plan reviewed using the template. Agreements and disagreements in the perspectives of participants were teased out within these triads. Following the completion of six within-case analyses findings were compared and contrasted across all cases, with the aim of drawing out key findings.

## Results

Table 1 summarises the characteristics of each of the six meso-level case study sites and the types and quantity of data generated in each. Illustrative quotations used below are labelled with the initial of the site pseudonym; then SM, SP, SU, CA or CC for senior manager, senior practitioner, service user, carer or care coordinator; and their unique number, e.g. B-SM-001 (Burgundy-Senior Manager-001).

### Survey results

#### Service users

**Fig. 3:**
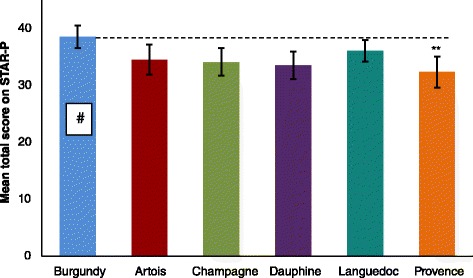
Mean total STAR-P score for service users ±95 % CI. Scoring range for the scale from 0 to 48. ** *p* = 0.01, # refers to the reference site

To explore cross-site differences of the perceptions of recovery-oriented practice, the therapeutic relationship and the perception of empowerment a series of one-way ANOVAs of all scales / subscales were conducted. Subsequent Tukey’s post-hoc tests demonstrated areas of significant difference between research sites on the STAR-P measure of therapeutic relationships. As Table 2 shows, there were significant differences across sites in the mean total STAR-P score (F (5, 429) = 3.45, *p* = 0.005), the positive collaboration subscale (F (5, 426) = 3.75, *p* = 0.002) and the positive clinician input subscale (F (5, 431) = 2.80, *p* = 0.017). This also shows that there were no substantive differences across the sites for the RSA (recovery scale) and ES (empowerment scale). Burgundy performed particularly well on the STAR-P scale, and if used as a reference site other sites can be considered in relation to it (see Fig. 3).

**Table 2:** Summary scores for service user responses to the RSA, STAR-P and ES scales

		Artois	Burgundy	Champagne	Dauphine	Languedoc	Provence
	One-way ANOVA Parameters	Mean (SEM)	Mean (SEM)	Mean (SEM)	Mean (SEM)	Mean (SEM)	Mean (SEM)
Recovery Self Assessment Scale (RSA)							
Life Goals	F(5, 394) = 0.65, *p* = 0.659	3.48 (0.12)	3.55 (0.13)	3.38 (0.97)	3.43 (0.14)	3.31 (0.11)	3.30 (0.13)
Involvement	F(5, 373) = 0.81, *p* = 0.543	2.89 (0.15)	2.96 (0.13)	2.70 (0.15)	2.93 (0.16)	2.66 (0.13)	2.86 (0.15)
Diversity of Treatment Options	F(5, 406) = 1.67, *p* = 0.139	2.99 (0.15)	3.06 (0.13)	3.05 (0.14)	3.21 (0.15)	2.70 (0.11)	2.91 (0.14)
Choice	F(5, 423) = 1.27, *p* = 0.277	3.66 (0.11)	3.65 (0.10)	3.66 (0.10)	3.69 (0.13)	3.72 (0.09)	3.39 (0.11)
Individually Tailored Services	F(5, 418) = 1.72, *p* = 0.129	3.27 (1.00)	3.34 (0.13)	2.95 (0.13)	3.23 (0.14)	3.04 (0.12)	2.89 (0.14)
Mean Total Score	F(5, 405) = 0.86, *p* = 0.509	3.27 (0.12)	3.33 (0.11)	3.13 (0.11)	3.31 (0.13)	3.12 (0.10)	3.10 (0.12)
Scale to Assess Therapeutic (STAR-P)							
Positive Collaboration	F(5, 426) = 3.75, *p* = 0.002 **	17.37 (0.76)	19.81 (0.57)	17.13 (0.70)	17.29 (0.79)	18.62 (0.52)	16.15 (0.76)
Positive Clinician Input	F(5, 431) = 2.80, *p* = 0.017 *	8.12 (0.40)	9.46 (0.28)	8.01 (0.36)	8.22 (0.37)	8.46 (0.29)	7.83 (0.40)
Non Supportive Clinician Input	F(5, 430) = 1.66, *p* = 0.142	8.90 (0.28)	9.23 (0.33)	9.09 (0.33)	8.02 (0.45)	9.14 (0.30)	8.53 (0.36)
Mean Total Score	F(5, 429) = 3.45, *p* = 0.005 **	34.51 (1.31)	38.49 (1.00)	34.09 (1.21)	33.53 (1.21)	36.07 (0.95)	32.33 (1.37)
The Empowerment Scale (ES)							
Self-esteem – self-efficacy	F(5, 428) = 0.78, *p* = 0.563	2.57 (0.09)	2.60 (0.09	2.50 (0.09)	2.63 (0.10)	2.60 (0.08)	2.73 (0.09)
Power-powerlessness	F(5, 422) = 0.81, *p* = 0.542	2.43 (0.06)	2.51 (0.06)	2.44 (0.06)	2.42 (0.08)	2.45 (0.06)	2.57 (0.05)
Community activism and autonomy	F(5, 422) = 0.32, *p* = 0.901	3.13 (0.05)	3.07 (0.07)	3.05 (0.07)	3.12 (0.08)	3.09 (0.05)	3.14 (0.06)
Optimism and control over the future	F(5, 431) = 1.36, *p* = 0.238	2.62 (0.08)	2.63 (0.07)	2.51 (0.07)	2.70 (0.09)	2.61 (0.07)	2.77 (0.08)
Righteous anger	F(5, 428) = 0.58, *p* = 0.718	2.34 (0.09)	2.24 (0.08)	2.32 (0.07)	2.31 (0.10)	2.21 (0.08)	2.35 (0.06)
Mean Total Score	F(5, 429) = 1.41, *p* = 0.221	2.62 (0.05)	2.62 (0.05)	2.56 (0.04)	2.64 (0.05)	2.62 (0.05)	2.73 (0.04)

* Significant at the *p* < 0.05 level ** Significant at the *p* < 0.01 level

#### Care coordinators

To explore cross-site differences of the perceptions of recovery-oriented practice a series of one-way ANOVAs were conducted for the mean RSA total score and the five RSA subscales for the care coordinators. As Table 3 shows, there was a significant difference between the research sites in the ‘Choice’ subscale (F(5,195) = 3.40, *p* = 0.006) but no significant differences in the mean total RSA scores or on the other four subscales. ‘Choice’ includes freedom from threats and coercion and being provided with support in the home, community, or workplace.

**Table 3:** Summary scores for the care-coordinator responses to the RSA scale

Recovery Self Assessment Scale (RSA)	One-way ANOVA Parameters	Artois	Burgundy	Champagne	Dauphine	Languedoc	Provence
		Mean (SEM)	Mean (SEM)	Mean (SEM)	Mean (SEM)	Mean (SEM)	Mean (SEM)
Life Goals	F(5, 195) = 0.71, *p* = 0.617	3.68 (0.12)	3.73 (0.11)	3.79 (0.09)	3.54 (0.15)	3.82 (0.11)	3.70 (0.09)
Involvement	F(5, 195) = 0.98, *p* = 0.429	3.01 (0.13)	2.91 (0.11)	2.92 (0.13)	2.99 (0.15)	3.23 (0.12)	2.87 (0.10)
Diversity of Treatment Options	F(5, 195) = 2.10, *p* = 0.068	2.96 (0.14)	3.23 (0.13)	2.94 (0.13)	2.98 (0.16)	3.24 (0.12)	2.74 (0.09)
Choice	F(5, 195) = 3.40, *p* = 0.006 **	3.76 (0.10)	3.92 (0.11)	3.70 (0.11)	3.46 (0.10)	4.04 (0.10)	3.58 (0.13)
Individually Tailored Services	F(5, 195) = 1.74 *p* = 0.126	3.18 (0.13)	3.10 (0.13)	3.11 (0.13)	3.49 (0.15)	3.42 (0.15)	3.42 (0.13)
Mean Total Score	F(5, 195) = 0.997, *p* = 0.421	3.35 (0.11)	3.41 (0.10	3.35 (0.11)	3.31 (0.13)	3.57 (0.11)	3.25 (0.08)

** Significant at the *p* < 0.01 level

#### Correlations between outcome measures

Pearson’s correlations were used to assess the within-group associations between questionnaire scales. Table 4 shows that there is a large positive correlation (*r* = 0.61, *n* = 409, *p* < 0.001) [34] between the RSA and STAR-P. Amongst service users, this indicates there is an association between their ratings of the recovery-oriented focus of organisations and ratings of the quality of relationships. There is a small-medium relationship between the RSA and ES (*r* = 0.20, *n* = 406, *p* < 0.001) and a small relationship between the STAR-P and the ES (*r* = 0.14, *n* = 431, *p* < 0.01).

**Table 4:** Correlation analysis of service user responses to the outcome scales

Measures	Parameters	Total sample
RSA and STAR-P	r	0.607
	Sig.	0.000 ^a^
	N	409
RSA and ES	r	0.204
	Sig.	0.000
	N	406
STAR-P and ES	r	0.138
	Sig.	0.004
	N	431

^a^ Correlation is significant at the 0.01 level

### Qualitative findings

#### Local context and developments

Analysis of interview and documentary data found that in all six sites, services had recently been or were still subject to reorganisation and moves towards greater integration of health and social care staff within teams. One site (Languedoc) appeared to be moving in a different direction with social workers removed from CMHTs to focus on social care assessments and personal budget applications. One senior manager said:

“*Very rarely you would find a social worker, because under the Section 75 agreement they’re no longer expected to be a care coordinator*” *[L-SM-001].*

This reportedly led to higher workloads within CMHTs and lower morale; this was also the only site where medical domination of services was cited as an issue. Greater uncertainty about developments was evident where organisational change had been particularly extensive, such as in Provence where services were being centralised and teams were working across wider age groups.

Some service changes were reportedly driven by austerity measures designed to reduce public expenditure (e.g. merging of assertive outreach or crisis teams with CMHTs, and reductions in social care services) or (in the English sites) by new legislation opening up care delivery to non-statutory providers. In the two Welsh sites, the new legislation placing CTPs on a statutory footing (the Mental Health (Wales) Measure) was described by senior staff as a driver for developments. One senior manager said the Measure had:

“…*provided the impetus for everything else that’s come*” *[B-SM-001].*

However, the impact of this on everyday practice was less evident. Integration of health board and local authority managers and teams was high on the agenda, although integration in Champagne appeared to be frustrated by continuing differences at the most senior levels and the absence of shared and accessible information (IT) systems. Frustrations with inflexible IT were articulated across all sites, as identified by this care coordinator:

*“The CPA computer system’s awful. It makes things a lot more difficult, and it’s not just with care plans, risk assessments, you have to lock them, and then you have to go and pull the whole thing through, you can’t just edit it, go in and edit it, so it’s really time consuming” [P-CC-002].*

Commitments to the idea of ‘recovery’ in all sites appeared frustrated by seemingly contrary policies (e.g. community treatment orders, which compelled people to accept services) and high levels of administrative demand. One senior manager observed:

“…*it’s almost like it’s [the CPA] been hijacked*” *[A-SM-002].*

#### Care planning and coordination

Major challenges were identified across sites in trying to make care planning and care coordination meet the very different needs of service users, practitioners, managers and service commissioners, as alluded to by this manager:

“…*obviously, you’re pleasing two beasts, performance commissioners, as well as trying to balance that with making sure your paperwork is service user friendly, which is challenging*” *[P-SM-002].*

In England, the CPA was reported to be administratively heavy, and insufficiently oriented towards recovery.

*“Paperwork for instance takes probably up to like 75 % of the care co-ordinator’s time… you probably see somebody here for an hour and [the paperwork]… ends up taking probably half of your day and that then is not useful at all… [because] that time you could have been using to see other people” [D-CC-002].*

Concerns were expressed that new general policies (e.g., the introduction of Clustering and Payment by Results, through which patients were grouped together for the purposes of commissioning services and releasing payments [38]) were resulting in less individualised care plans. This senior practitioner was particularly critical:

“…a*nother layer of paperwork that needs to be completed and another target that needs to be met*” *[D-SP-005].*

In Wales, the Measure was seen as very important but there was no consensus on its actual impact. Views regarding the CTP were mixed. In both sites, it was agreed the CTP template provided a structure, but was also seen by some staff as reductionist or simply bureaucratic:

“…*in most cases, they see it [the CTP] as a paper exercise*” *[B-SP-003].*

Care coordinator caseloads reportedly ranged from 25 (Artois) to 40 or more (Champagne, Dauphin, Languedoc) or even 50 (Provence), and in most sites these were increasing due to growing demands and tighter staff budgets. The care coordinator role was held by any mental health professional; most often mental health nurses, social workers and occupational therapists, less often psychologists, and rarely psychiatrists. In one site (Artois) ‘non-professional’ staff were able to act as care coordinators. Overall, the role was seen as ‘generic’, but also a complex and responsible one, as articulated by these senior managers and practitioners:

*“…we do something very similar, whether you’re a social worker, OT or nurse*” *[B-SM-002].*

“…*the role is so complex and there’s so much uncertainty*” *[D-SP-001].*

The desire to be responsive and provide a more personalised approach to care coordination was often frustrated by the lack of capacity within a team’s or individual’s caseload.

“…*whatever discipline you are if you’re that person’s care co-ordinator the expectation would be that you would do everything a care co-ordinator is expected to do […] sometimes people will be allocated a care coordinator because of availability and capacity rather than best fit. That can be a problem*” *[A-SP-005].*

The introduction of electronic documentation appeared to bring some benefits in terms of accessing and sharing information but also unwieldiness and a greater distancing from service users, as working collaboratively with people on care plans in their homes paradoxically became more difficult. This practitioner explains:

*“…extra demands of doing the [CTP] paperwork within a specific time… the job has become much more desk orientated, rather than face to face work*” *[B-SP-003].*

Efforts to increase the involvement of families and carers was mentioned occasionally but more often it was the challenges and continuing tensions around issues such as service users’ consent and confidentiality that were mentioned, or uncertainty around how to involve people.

There were varied experiences of care planning and care coordination from the viewpoints of service users, carers and care coordinators across all six sites with no distinct differences between sites identified. At best, care was planned in a structured and collaborative way with clear communication and opportunities for service users to influence and feel some ownership of the process.

*“…it’s very much [the service user’s]… as much as possible we put the onus on the patient” [C-CC-005].*

“…*a certain number of our care plans are written in the first person because that’s really the way that we should be doing it with CPA because it’s all about the service use*r” *[D-SM-002].*

At worst, service users said they felt insufficiently involved, or that their care was planned as an obligatory task and in ways which were confusing and rigid.

*“…the form is a prompt for them to make sure they’ve covered everything rather than a personalised summary for me…” [A-SU-002].*

Risk was consistently seen as central to the CPA/CTP process by senior staff across all six sites, as evident in these quotes:

“…*the topmost heading…irrespective of anything else really*” *[A-SP-002].*

“…*very much at the forefront of what we do…safety and risk, rightly or wrongly drives what we do, very much, and a lot of the time*” *[C-SP-002].*

“…*the thing that we score best on in…audits is the risk assessment, simply because people think that that’s more important…and to some extent they’re probably right*” *[L-SM-002].*

Similarly, safety and risk were paramount for care coordinators, but service users and carers reported that they were hardly involved in risk assessment process and many staff explicitly expressed concerns over sharing their views, or those of their colleagues, with service users.

*“…[the risk assessment is] one thing… you never discuss with service users just in case it alarms them*” *[B-SM-001].*

Consequently, service users and carers were often unaware of risk assessments being undertaken.

*“…[at home] nobody’s checked up on me or anything” [C-SU-005].*

Documented care plans were useful for staff and were recognised as having to be created, and had value as records of what has been decided and for including contact details and, sometimes, crisis and contingency plans. However, for the majority of participants care plans were not highly valued, and were not seen as active documents; many care coordinators and service users did not routinely refer to care plans once they had been created. Many service users did not have care plans, or had received and quickly discarded them. Others filed them, never to be looked at again:

*“[I] stick them in a drawer somewhere” [A-SU-002].*

Some care coordinators mentioned the development of first person care plans but service users rarely if ever mentioned this. Carers had varied experiences within sites; some felt involved in the process, but many were not engaged with care planning and coordination, with one saying:

*“…it makes no difference to us” [B-CA-004].*

Some did not see this as problematic, seeing it as something that occurred primarily between the service user and their care coordinator.

#### Recovery

Amongst senior personnel there was some clear articulation of what recovery and recovery-focused values might be, but varying degrees of sign-up to recovery and frequent talk of resistance amongst some staff.

“…*providing hope, and delivering a service that actually makes that person feel hopeful that they can recover in the first instance… [and] that they will get out of life what they want to get out of life while living with an illness*’ *[C-SP-001].*

*“[I] have not heard the word recovery mentioned, not even once*” *[D-SP-001].*

In Languedoc for example, there was a broad understanding and evidence of a Recovery College, co-produced care plans and some use of recovery tools (e.g. Wellness Recovery Action Plans (WRAP) [39], Recovery Star [40, 41]) but a reported lack of passion amongst senior managers and medical staff content to retain more traditional approaches.

“…*when everybody started to talk about recovery, people thought it’s something that we do anyway…I don’t think that there’s always been appreciation of the depth of it*”, *[and how]* “*people in some respects underestimated the level of investment that you have to make as an organisation and as a practitioner to work in a true recovery focused way*” *[L-SM-001].*

Some resistance amongst medical staff and some older nursing staff was also reported in Provence, alongside a belief that ‘not all service users will recover’, as suggested by a senior practitioner here:

“*I’ve got people on care packages and the actual wording is,* ‘*please demonstrate how they’re going to require less input next year as they will improve’ and you just think that they won’t. This recovery is actually a bit of a misnomer*” *[P-SP-002].*

Nonetheless, managers in Provence also cited the introduction of peer workers and greater use of ‘recovery language’. In Artois, recovery was seen as a ‘bridge between medical and social models’ and also something ‘we have always been doing’. It was also seen as about discharging service users reluctant to move on. Barriers to the implementation of a greater focus on recovery in Artois included the need for more staff, more time, improved IT systems and a stronger organisational commitment. Senior staff in Dauphine acknowledged that the move towards recovery had been fairly recent and had been met by resistance amongst some staff. However, there was a drive to increase the use of personal budgets and help with obtaining employment was now much more commonplace.

In Wales, the Measure had placed recovery high on the agenda for senior staff, with good understandings of recovery seen in Champagne but more mixed understandings of recovery expressed in Burgundy. For some in Champagne, the CTP did not have a clear focus on recovery and it was felt that training was needed for both staff and service users to bring about a change in culture.

As with care planning, service users, carers and care coordinators had varied views and experiences in relation to recovery across all sites, with the term itself often having different meanings for different people. Different views between professional groups were also mentioned. For some care coordinators it was even seen as unhelpful or deceptive as it appeared more about encouraging the discharge of service users from caseloads. Across sites, many service users used terms commonly found in recovery literature, such as choice, independence, hope, fulfilment. But for many it was primarily about managing and coping better with their illness, as explained by this service user:

*“…finding the best way to live with whatever you’re going through and adapting better to it” [A-SU-002]*

Very few talked about recovery as a ‘journey’. There were no clear views that care planning helped recovery, unless care plans included practical steps, or where they helped service users to accept or talk about their mental health, as suggested by this service user and carer:

“*I think the thing I found most helpful was having a structure to… my care plan and a weekly timetable to follow. So it’s helped me stay stable and build a structure using weekly timetables to add full range of activities” [P-SU-001].*

*“You can write anything on a piece of paper but if nothing is getting done practically, it doesn’t mean anything” [D-CA-002].*

Some service users and carers said that strengths were acknowledged. For service users and carers in particular, conversations and relationships were identified as being far more important than care plans in promoting recovery, along with family and friends.

Even within single sites there was variable use of, experience in, and enthusiasm for recovery tools. At best, Wellness Recovery Action Plans (WRAPs) [39], which are a structured system for monitoring and managing distressing symptoms and unhelpful behaviour patterns, are seen as helpful and more personalised than care plans:

“*Nobody can write a WRAP plan for me but a care plan can be written without someone… it’s the WRAP plan that belongs to me and it’s the care plan that belongs to the professionals” [A-SU-002].*

But some care coordinators worry about the extra work required. Other care coordinators say they have always worked in a recovery-focused way, and what hindered them were organisational targets and issues such as adversity to risk, documentation, limited resources, and ‘firefighting’ (i.e. responding to emergent priorities).

#### Personalisation

Across the case study sites, personalisation was not as consistently understood as the concept of recovery amongst senior personnel, and definitions tended to include person-centred care plans and the use of first-person terminology in care plans (Artois); placing the person at the heart of social care (Artois); and the use of personal budgets (Artois, Dauphine) and direct payments (Champagne) to purchase aspects of care and support.

In relation to personal budgets, a relatively new approach which devolves money for health care to the individual using services, allowing them to purchase individualised care [42], there was a clear lack of uniformity. This was also the one area where there was a marked difference between the two countries, reflecting the different policy emphases. In England, personalisation is portrayed in policy documents as a means of ‘giving people greater choice and control over their care and treatment’ [43] (pp. 32–3). In Wales, an Independent Commission on Social Services rejected this view of personalisation, stating that ‘the label ‘personalisation’ has become too closely associated with a market-led model of consumer choice’ [44] (pp15). Some sites saw the use of personal budgets as a key tool in the move towards a recovery-focus and personalisation of care (e.g., Dauphine, Languedoc, Provence). In both sites in Wales, there was much less emphasis on the use of personal budgets, which were seen very much as part of a creeping ‘marketisation’ of healthcare and as a consequence subject to more resistance.

Even where personal budgets were promoted, it was recognised that they were accompanied by extremely heavy administrative loads which took practitioners away from face-to-face contact with service users and their families (Artois, Dauphine, Languedoc).

*“I suppose often care co-ordinators, myself included, sometimes we get slightly panicked when people talks about personalisation because we think… that’s going to generate a lot of work” [D-CC- 004].*

There were also often severe delays in actually receiving funds, which impacted on service users, staff and relationships (Dauphine). Elsewhere, service users often failed to meet the strict criteria against which they were appraised, or were reluctant to make a contribution (Languedoc). Senior staff also spoke of there being tensions when service users were unwell and that talk of a more personalised approach could ‘raise expectations’ that could not always be met (Champagne).

There was also an articulation of some of the tensions that exist in the supposed move towards greater personalisation, with compulsory treatment orders (CTOs) (Artois) and clustering (a system of grouping patients, as a precursor to releasing payments from commissioners to care providers [38]) cited as counterpoints (Dauphine).

“…*there’s tensions about how it works within mental health particularly with CTOs being a big thing*” *[A-SP-004].*

*“…[clustering is] not personalised and I’m not sure why we’re moving in that direction” [D-SP-005].*

Changes to health care provision in England following the passing of the Health and Social Care Act 2012 had resulted in commissioners being able to purchase services from ‘any qualified provider’ [45], which for some had raised issues around sharing information and issues of confidentiality (Dauphine). One senior manager suggested that:

‘…*whilst very attractive and trendy at the moment, I think after a couple of homicide reports or suicide reports, high profile, that says people can’t talk to each other, people don’t talk to each other because they work for different organisations, I think there’ll be demand for people to be brought back into one organisation*’ *[A-SM-002].*

Uniquely, in Languedoc, under an agreement made between the local authority and the Trust, social workers had been removed from CMHTs to focus on assessments and processing of direct payments and personal budgets. The aim was to increase the move towards the use of personal budgets by service users to purchase care and support, which was seen as a key tool in the move towards personalisation of services. It appeared that while the success of this approach was still to be determined, the impact on remaining staff within CMHTs was less than positive with caseloads and workloads increasing with the loss of the social workers from the teams.

Service users and carers described their own care as being personalised, or as very much not personalised, depending on personal experiences, with no distinct differences between sites emerging.

“*It means putting the person at the centre of what you do, so not forgetting that you’re dealing with human beings and that every human being is an individual and that what works for one person won’t necessarily work in exactly the same way for another person” [A-SU-002].*

“…*tailoring the plan around me” [B-SU-001]*

*“…[it’s] rubbish… what care? What personalisation?” [B-CA-003].*

Personalisation was constrained by lack of resources and availability of local services, by service users not always being aware of options available to them, and by service users deferring to professionals. Gaps were observed between the ideal and the reality, with staff reporting high administrative workloads and the complexity of actually accessing and using personal budgets.

“…*absolutely dire [because they turn a] simple process…into an absolute mass of paperwork […]*” [L-SP-002].

As with recovery, some care coordinators reported that personalisation can raise unrealistic expectations:

*“I think again if you give someone complete full reins to go ahead and design their care plan it just becomes unrealistic so I think it’s just about being realistic all the time… And being realistic isn’t, I don’t think, it’s not a bad thing. It’s about what’s available to us, and that’s not because I don’t want to help my client get whatever they want but it’s what’s available to us as a service and resources that are available to us” [P-CC-002].*

## Discussion

The aim for this cross-national study was to identify and describe the factors associated with collaborative, personalised, and recovery-focused care planning and coordination in community mental health services. We sought to address this aim by investigating the views and experiences of care planning and coordination of service users, informal carers, care coordinators, senior practitioners and managers; measuring service user perceptions of recovery-oriented practices, therapeutic relationships and perceptions of empowerment and staff perceptions of recovery oriented practices. Finally we compared experiences between sites and between England and Wales to explore organisational and national policy-level influences.

Data were generated in the context of policy divergence across England and Wales. However, comparisons of survey results across sites, and analysis of micro-level interview data, raise questions over the extent to which changes such as Wales’ Mental Health Measure are being ‘felt’ by service users at the frontline. Only cautious claims of significant differences in scores on the three measures across the six sites can be made, and such differences as can be detected followed no national pattern across England and Wales. Differences in the degrees of service user involvement in care planning qualitatively described by participants varied within and across sites, again with no discernible national pattern.

Large-scale policy in both England and Wales places considerable emphasis on both the process and the product of care planning. However, across all sites participants described various difficulties with care planning, and with the content and utility of completed care plans as documents. We found evidence of care planning and coordination being shaped by large-scale imperatives to adhere to mental health-specific law and policy but also to other policies such as clustering and payment by results, to commissioners’ demands for monitoring data and to services managers’ needs to review and improve professional performance. For many, care planning and coordination was primarily about the assessment and management of risk, involving the construction of professional judgments made with little collaboration with people using services. The implication of this being that professional concerns about risk and safety may take precedence over broader everyday risks experienced by people using services [46]. In the face of competing macro and meso-level pressures, at the micro-level we heard of care plans being developed and then forgotten about by service users and practitioners unsure or unable to make active, day-to-day, use of them. Frequently, service user participants were unable to talk knowingly about the way their care plans were produced, or subsequently used.

Across all sites we found evidence that austerity, as an explicit macro-level response to economic collapse [47], was being felt. Caseloads were described as rising and services in many areas had undergone major reconfiguration in an effort to create ‘efficiencies’ or reduce costs. Opportunities to match care coordinators to service users on the basis of need were challenged by practical contingencies including care coordinators’ caseloads being full. If care coordinators are likely to become *de facto* providers of most care, as some participants said was the case, then the degree of fit between user and practitioner is an important consideration. Here, ‘fit’ refers to the particular constellation of skills and knowledge possessed by staff reflecting, to some degree, professional backgrounds but also the degree of ‘fit’ at the interpersonal level. Teams where staff are overworked have less capacity to make optimal alignments between people using services and those coordinating and providing these [48]. Such restrictions are distinctly at odds with new legislation offering greater choice to mental health service users [49]. Care coordinators were not universally professionally trained, nor necessarily trained in care coordination or to involve people in their care, as reported elsewhere [50], raising further questions about preparedness to take on a significant and challenging role.

Recovery is a theme found in macro-level mental health policy equally across England and Wales, and as a concept is well discussed in both the literature [51] and within service user research and activist circles [52]. Its filtering into practice has been a less consistent affair, and in parallel to the literature [53] we encountered little in the way of shared understanding of recovery in our study sites. Aspirations that recovery provides a cultural and values-based approach to improving mental health care may be some way from being realised. Risk, in contrast, was described as driving processes of care planning and coordination more than any other consideration.

Personalisation, as a macro-level idea found in recent policy and guidance [54], was understood (if at all) in different ways by different people. Large-scale ambitions that care be uniquely tailored to individuals’ needs clashed (for example) with micro-level evidence that setting up personal budgets was bureaucratic, exceptionally time-consuming and frequently obstructive. There was also evidence of major difficulties in ensuring personal budgets were agreed, and that once agreed that monies were received. Lengthy delays created frustrations for service users and staff alike and there were reports of these frustrations harming the therapeutic relationship. There is an increasing literature on personal budgets that suggests they may be a force for greater choice, flexibility, control and empowerment [42], but unless the processes are improved our data suggests they may appear as suspiciously like another laborious, hugely bureaucratic process – rather like the CPA was on its introduction [55].

In contrast, close correlations were found between scores for therapeutic relationships provided by people using mental health services, and the recovery-oriented nature of those services. People using services value the relationships they have with workers [50, 56, 57] and our research interviews reaffirmed this. The relationship with the worker was seen as one of the few constants in an ever-changing landscape of health and social care provision.

## Limitations

Within each sub-group of service users, carers, practitioners and managers, there may have been an element of self-selection or inherent biases not immediately apparent to the researchers. There appears to be a wide selection of professions and viewpoints identified amongst the staff interviews but the service user sample was weighted more towards those with long-standing contact with services. This may have been reflected in more limited experiences of a more recovery-focused approach from clinicians who may feel longer-term services users are less likely to respond to a focus on recovery. Similarly, such a population may have different expectations of care coordinators and mental health services than a younger sample less habituated to the familiarities of mental health service delivery.

There was a moderate level of missing data for the RSA scale completed by service users, possibly due to some of the difficult language used. As a consequence, more detailed analysis of co-variations within the data was restricted by lack of power. The RSA was selected for use after consideration of several organisational-focused measures of recovery. Nonetheless, this was not a satisfactory measure as too many participants found some of the language and North American terminology unfamiliar and unclear. Adaptation and re-validation of this measure to a British population or the identification of a more suitable measure would be recommended for future studies.

## Conclusions

The results of this cross-national, multi-site mixed methods study suggest that there exists a gap between the macro-level national policy aspirations for recovery focused, personalised care planning and coordination and the meso/micro-level ‘street-level’ practices and everyday experiences of service users, carers and care coordinators. Of particular concern was evidence of a perhaps widening discrepancy between policy and practice and the indications of an emergent cynicism amongst participants as recovery concepts and ideals are subverted by higher-order organisational needs, directives and ends. There is a serious risk that the hope and optimism that recovery approaches can offer mental health services is being dampened and perhaps snuffed out by the ‘re-conceptualisation of recovery’ at a macro-level.

Amongst participants in this study within and across the six sites, there was a lack of consensus about what recovery means. This may be expected with such a relatively nascent and contested concept [51]. However, a loss of focus and legitimacy at a time when services are and will continue to be under enormous pressure to respond to the increasing demands placed upon them at a time of continued austerity [47, 58] is worrying and could have serious ramifications for the engagement, safety and wellbeing of local populations and communities and the retention of top quality staff. Mental health service commissioners and providers need to ensure there is clarity and consistency in establishing and communicating with partners and recipients of services what is meant by recovery. They also need to ensure the aims and operations of the organisation are designed to support staff and service users in realising that vision.

Care planning itself was seen by care coordinators and managers as a useful way of recording and evidencing plans and actions but was largely deemed irrelevant thereafter by most frontline staff and the majority of service users. Yet the processing, completing, updating and uploading of care plan documentation is reported to require considerable time and energy away from direct contact with services users, families and wider networks whilst appearing to play a minimal role in aiding recovery. Information technology may provide some assistance in accessing and sharing information but paradoxically appears more often to require even yet more time away from the service user as care coordinators grapple with inflexible, unwieldy systems and, from the service user’s viewpoint, de-personalised outputs.

Issues of safety and risk go hand-in-hand with mental health service delivery, perhaps more than in any other area of healthcare. Our data showed clearly that for managers, senior practitioners and front-line clinicians, risk assessment and management is central to their work and a key component in care planning and coordination. Yet for the majority of service users and some carers interviewed in this study, this was far less evident and there was a clear disjuncture between these experiences. Most service users did not feel their safety had necessarily been considered, nor that perceptions of their risk towards others been discussed with them. In order to provide genuinely personalised, recovery-focused care planning and to ensure the safety and wellbeing of all, attention must be given to how greater openness, partnership working and shared decision-making can be developed in this important area.

After 25 years of the CPA (and its more recent CTP sibling) and repeated accounts of bureaucratic overload, it is time for innovative, flexible, genuinely more person-centred solutions to this dilemma. It is clear from service users and carers in this study that the key instrument in helping and enabling people towards recovery is the therapeutic relationship with empathic, respectful, skilful care coordinators and wider family and social support networks. The allocation or choice of care coordinator and care planning processes must be redesigned to support not hinder that. Wellness Recovery Action Plans and similar approaches, as often discussed in our data, may provide a more individualised and recovery-focused method that merits more detailed investigation, especially in light of recent evidence [59]. However, introducing these *in addition* to existing procedures will likely create greater stress and resentment, rather than solutions.

## Ethics approval and consent to participate

The study received a favourable NHS Research Ethics opinion from the National Research Ethics Service (NRES) Yorkshire and The Humber Committee – Sheffield (Ref: 13/YH/0056A). A major amendment was approved to allow a reminder letter to be sent to service users for the questionnaire component and for the interview invitation letter to include information about service user and carer participants receiving a small payment on completion of interview. Research governance approvals were also secured from each of the six participating NHS care provider organisations. All participants provided written consent to participate.

## Consent for publication

Not applicable.

## Availability of data and materials

Data can be provided on request.

## Additional files

Additional file 1: Service user semi-structured interview COCAPP, interview schedule. (DOCX 124 kb) Additional file 2: Care coordinator semi-structured interview COCAPP, interview schedule. (DOCX 123 kb) Additional file 3: Carer semi-structured interview COCAPP, interview schedule. (DOCX 125 kb) Additional file 4: Managers semi-structured interview COCAPP, interview schedule. (DOCX 120 kb)

## Abbreviations

ANCOVA: Analysis of Covariance
ANOVA: Analysis of Variance
CMHT: Community Mental Health Team
CPA: Care Programme Approach
CTO: Community Treatment Order
CTP: Care and Treatment Planning
ES: Empowerment Scale
IT: Information Technology
LEAG: Lived Experience Advisory Group
LHB: Local Health Board
NRES: National Research Ethics Service
PAG: Project Advisory Group
REC: Research Ethics Committee
RSA: Recovery Self-Assessment
STAR-P: Scale to Assess the Therapeutic Relationship – Patient Version
SUGAR: Service User and Carer Group Advising on Research
UK: United Kingdom
WRAP: Wellness Recovery Action Plan

## Footnotes

1 Service user is used in this article to denote a patient or user of psychiatric and mental health services. A carer is a family member or friend providing some level of emotional or practical support to the service user.

## References

[1] Ham C, Baird B, Gregory S, Jabbal J, Alderwick H (2015). The NHS under the coalition government. Part one: NHS reform.

[2] Longley M, Riley N, Davies P, Hernández-Quevedo C (2012). United Kingdom (Wales): Health system review. Health Syst Transit.

[3] Hannigan B, Coffey M (2011). Where the wicked problems are: the case of mental health. Health Policy.

[4] Department of Health (1990). The care programme approach for people with a mental illness referred to the specialist psychiatric services. HC(90)23/LASSL(90)11.

[5] Department of Health (2008). Refocusing the care programme approach: Policy and positive practice guidance.

[6] Welsh Assembly Government (2003). Mental health policy guidance: The care plan approach for mental health service users.

[7] Welsh Government (2012). Code of practice to parts 2 and 3 of the Mental Health (Wales) Measure 2010.

[8] Anthony WA (1993). Recovery from mental illness: The guiding vision of the mental health service system in the 1990s. Psychiatr. Rehabil. J..

[9] Welsh Government (2012). Together for mental health: A strategy for mental health and wellbeing in Wales.

[10] Dieterich MIC, Park B, Marshall M (2010). Intensive case management for severe mental illness. Cochrane database of systematic reviews.

[11] Burns T, Yiend J, Doll H, Fahy T, Fiander M, Tyrer P (2007). Using activity data to explore the influence of case-load size on care patterns. Br J Psychiatry.

[12] Simpson A (2005). Community psychiatric nurses and the care co-ordinator role: squeezed to provide ‘limited nursing’. J Adv Nurs.

[13] Commission for Healthcare Audit and Inspection (2007). No voice, no choice: A joint review of adult community mental health services in England.

[14] Wales Audit Office (2011). Adult mental health services: follow up report.

[15] Hewitt J, Coffey M (2005). Therapeutic working relationships with people with schizophrenia: Literature review. J Adv Nurs.

[16] McCabe RP, Priebe S (2004). The therapeutic relationship in the treatment of severe mental illness: A review of methods and findings. Int J Soc Psychiatry.

[17] De Leeuw M, Van Meijel B, Grypdonck M, Kroon H (2012). The quality of the working alliance between chronic psychiatric patients and their case managers: process and outcomes. J Psychiatr Ment Health Nurs.

[18] Yamashita M, Forchuk C, Mound B (2005). Nurse case management: Negotiating care together within a developing relationship. Perspect Psychiatr Care.

[19] Kondrat DC, Early TJ (2010). An exploration of the working alliance in mental health case management. Soc Work Res.

[20] Simpson A, Hannigan B, Coffey M, Jones A, Barlow S, Cohen R, Všetečková J, Faulkner A, Haddad M (2015). Study protocol: cross-national comparative case study of recovery-focused mental health care planning and coordination (COCAPP). BMC Psychiatry.

[21] Simpson AHB, Coffey M, Jones A, Barlow S, Cohen R, Všetečková J, Faulkner A. Cross-national comparative mixed-methods case study of recovery-focused mental health care planning and co-ordination: Collaborative Care Planning Project (COCAPP). Health Serv Deliv Res. 2016;4(5). 26866206

[22] Byrne DS (1998). Complexity theory and the social sciences: An introduction.

[23] Creswell J (2009). Research Design: Qualitative, quantitative and mixed methods approaches.

[24] Stake RE (1995). The art of case study research.

[25] Yin RK (2014). Case study research: Design and methods.

[26] Kline P (2000). Handbook of psychological testing.

[27] O’Connell M, Tondora J, Croog G, Evans A, Davidson L (2005). From rhetoric to routine: assessing perceptions of recovery-oriented practices in a state mental health and addiction system. Psychiatr Rehabil J.

[28] Mcguire-Snieckus R, McCabe R, Catty J, Hansson L, Priebe S (2007). A new scale to assess the therapeutic relationship in community mental health care: STAR. Psychol Med.

[29] Rogers ES, Chamberlin J, Ellison ML, Crean T (1997). A consumer-constructed scale to measure empowerment among users of mental health services. Psychiatr Serv.

[30] Gould D (2012). Service users’experiences of recovery under the 2008 Care Programme Approach.

[31] Simpson A, Barlow S, Cox L, Jones J, SUGAR (2014). Adding SUGAR: Collaborating with service users and carers in mental health nursing research. J Psychosoc Nurs Ment Health Serv.

[32] Corp IBM (2012). IBM SPSS Statistics for Windows; Version 21.

[33] Breakwell GM (2012). Research methods in psychology.

[34] Cohen J (1992). A power primer. Psychol Bull.

[35] QSR International Pty Ltd (2012). NVivo qualitative data analysis software; Version 10.

[36] Ritchie J, Lewis J, Nicholls CM, Ormston R (2013). Qualitative research practice: A guide for social science students and researchers.

[37] Ritchie J, Spencer L, Bryman A, Burgess R (1993). The Analysis of Qualitative Data: An approach to analysis for applied social policy research. Analyzing qualitative data.

[38] Clark M (2011). Mental health care clusters and payment by results: considerations for social inclusion and recovery. Ment Health Soc Incl.

[39] Copeland ME (1997). WRAP: Wellness recovery action plan.

[40] MacKeith J, Burns S, Facey E, Johnson J (2010). Mental Health Recovery Star: Organisational Guide.

[41] McKeith J, Burns S, Onyemaechi I, Okonkwo N (2010). The recovery star: User guide.

[42] Alakeson V, Perkins R (2012). Recovery, personalisation and personal budgets.

[43] Do H (2011). No health without mental health: a cross-government mental health outcomes strategy for people of all ages.

[44] WAG (2011). Sustainable social services for Wales: A framework for action.

[45] Timmins N (2012). Never again? the story of the health and social Care Act 2012: A study in coalition government and policy making.

[46] Coffey M (2012). A risk worth taking? Value differences and alternative risk constructions in accounts given by patients and their community workers following conditional discharge from forensic mental health services. Health, Risk & Society.

[47] Docherty M, Thornicroft G (2015). Specialist mental health services in England in 2014: overview of funding, access and levels of care. Int J Ment Heal Syst.

[48] Hannigan B, Allen D (2011). Giving a fig about roles: policy, context and work in community mental health care. J Psychiatr Ment Health Nurs.

[49] Department of Health (2014). 2014/15 Choice Framework.

[50] Bee P, Playle J, Lovell K, Barnes P, Gray R, Keeley P (2008). Service user views and expectations of UK-registered mental health nurses: A systematic review of empirical research. Int J Nurs Stud.

[51] Leamy M, Bird V, Le Boutillier C, Williams J, Slade M (2011). Conceptual framework for personal recovery in mental health: systematic review and narrative synthesis. Br J Psychiatry.

[52] Kalathil J (2011). Recovery and Resilience: African, African-Caribbean and South Asian women’s narratives of recovering from mental distress.

[53] Le Boutillier C, Leamy M, Bird VJ, Davidson L, Williams J, Slade M (2011). What does recovery mean in practice? a qualitative analysis of international recovery-oriented practice guidance. Psychiatr Serv.

[54] Confederation NHS (2015). Personal budgets in mental health: key points on implementation.

[55] Simpson A, Miller C, Bowers L (2003). The history of the Care Programme Approach in England: Where did it go wrong?. J Ment Health.

[56] Adam R, Tilley S, Pollock L (2003). Person first: What people with enduring mental disorders value about community psychiatric nurses and CPN services. J Psychiatr Ment Health Nurs.

[57] Coffey M, Higgon J, Kinnear J (2004). “Therapy as well as the tablets”: An exploratory study of service user views of community mental health nurses (CMHNs) responses to hearing voices. J Psychiatr Ment Health Nurs.

[58] Nuffield Trust (2012). A decade of austerity? The funding pressures facing the NHS from 2010/11 to 2021/22.

[59] Cook JA, Copeland ME, Jonikas JA, Hamilton MM, Razzano LA, Grey DD, Floyd CB, Hudson WB, Macfarlane RT, Carter TM (2012). Results of a randomized controlled trial of mental illness self-management using Wellness Recovery Action Planning. Schizophr Bull.

