# Supplementary material for: Recovery-focused care planning and coordination in England and Wales: a cross-national mixed methods comparative case study
Source: BMC Psychiatry. 2016 May 16;16:147. doi: 10.1186/s12888-016-0858-x (PMC4868048; doi:10.1186/s12888-016-0858-x)
Supplement: Additional file 1: — Service user semi-structured interview COCAPP, interview schedule. (DOCX 124 kb) [file 12888_2016_858_MOESM1_ESM.docx]

# COCAPP – Collaborative Care Planning Project

**Semi-Structured Interview Schedule**

**Service User/Patient Version 2: 08.02.2013**

Introduce yourself and explain nature of the study:

**Hi. My name is XXXX. Thank you for meeting with me today.**

**You kindly agreed to take part in the COCAPP research project and I am here today to ask you a few questions about your experience of care planning and coordination. It should take about 45 minutes at most. There are no right or wrong answers. We just want to know what you think about the way your care has been planned and coordinated.**

Remind the person that they have already given their consent to be interviewed and check that they are still OK with that. Remind them their name will not be used and they will not be identified in any way. They may stop at any time.

Check digital recorder and microphone are working and sound levels are adequate.

**I am just going to read out the code number for you in this study so that your name can be left out of it and the interview remains anonymous.**

*Read out Participant Code and Date.*

**1. Can you tell me how your care is planned by the Community Mental Health Team?**

*Prompts:*

- - *Do you have a written care plan? Is it working for you?*
  - *What did you find helpful? Less helpful?*
  - *Do you understand your care plan? Would a care plan in different formats be helpful (e.g. as a phone app?)*
  - *Are you aware of your care being planned?*
  - *How are you involved in the planning of your care?*
  - *What would help you to be more involved?*
  - *Does your care plan include a focus on your abilities, assets, skills, strengths? If so, could you give me some examples?*
  - *Do you feel ownership of your care plan – is it yours (or the service’s plan for you)?*
  - *What is important for you?*
  - *When and how often do you refer to your care plan?*
  - *Some actions in your care plan may be about things for you to do – how helpful is that?*

**2. Can you tell me about what happens when your care is reviewed?**

*Prompts:*

- *Do you have care review meetings? How helpful are they?*
- *What did you find helpful? Less helpful? Do you have enough time?*
- *Who was involved in those reviews?*
- *Do you have any choice about the timing, venue or who chairs the meeting?*
- *How were you involved? Could you contribute?*
- *Were your views listened to? Are your wishes and preferences taken on board?*
- *What would help you to be more involved?*
- *Have you had any experience of meetings with your care coordinator to prepare for review meetings or the use of a prompt list?*

**3. Please tell me about your Care Coordinator**

*Prompt:*

- *How did you first meet him/her?*
- *Do you have a good relationship with him/her?*
- *How long have you had this care coordinator? Have you had others?*
- *Was that similar or different?*
- *What did they do well/less well?*
- *Do you feel able to be open/express your fears with your care coordinator? Do you feel you trust your care coordinator?*
- *Are you aware of your care being coordinated? What does that mean to you?*

**4. What sort of support do you get from the Care Coordinator?**

*Prompt:*

- *Do you meet up? Does s/he phone you at all? Anything else?*
- *What is most helpful? What do they help you with?*
- *Are there things you would like more help with? What sort of things?*
- *How often do you see him/her?*

**5. Would you have liked to have had more of anything?**

*Prompt:*

- *What sort of thing would you have liked the Care Coordinator to have done more?*

**6. Was there anything that you didn’t like about the ways your care was planned?**

*Prompt:*

- *Was there anything that was unhelpful? Intrusive?*

**7. Did you have help or support from other workers?**

*Prompt:*

- *Like who? Social worker? Mental Health Nurse? Psychologist? Occupational therapist? Support worker?*
- *What has that been like?*
- *Does there appear to be communication between these different workers?*
- *Is your care coordinator involved in that?*
- *What information have you been given about other forms of support (e.g. support groups, peer support, user groups)?*

**8. Lots of people talk about Recovery in mental health nowadays – what does the term**

**Recovery mean to you?**

*Prompt:*

- *Thank you, that’s helpful. For many people, Recovery is generally seen as a personal journey ... one that may involve developing hope, a secure base and supportive relationships, being more in control of your life and care, social inclusion, how you develop coping skills, and self-management ... often despite still have symptoms of mental illness etc [ask next question]*

**9. How has the planning of your care helped with your Recovery?**

*Prompt:*

- *Have you been encouraged to develop a Personal Recovery Plan?*
- *A Wellness Recovery Action Plan (WRAP)?*
- *Have there been things that have helped your Recovery?*
- *Are there things you think might have helped your Recovery?*
- *Are your achievements recognised? If you made progress is it recognized, valued and recorded?*

**10. Another term that is being used a lot is ‘Personalisation’ - what does the term**

**‘Personalisation’ mean to you?**

*Prompt:*

- *Thank you, that’s helpful. For many people this term is often seen as putting service users firmly in charge of their care and support and that care is designed with their full involvement and tailored to meet their own unique needs. [ask next question]*

**11. Do you think your care and treatment was personalised?**

*Prompt:*

- *Did you think your care was tailored towards you and your individual needs?*
- *What does personalised care mean to you?*
- *Could you give me an example of where you think your care was personalised?*
- *In what way was it not focused on you as an individual?*
- *Do you feel in charge of your care and support?*

**12. Do you feel your safety has been considered in your care planning and coordination?**

*Prompts:*

- *How has your safety been addressed in your care plan or by your care coordinator?*
- *Have any other aspects of safety or risk been discussed with you?*

**13. Is there a family member or friend that provides you with support?**

*Prompt:*

- *Who is that? Are there others?*
- *Was s/he involved in the planning of your care? In what way?*
- *Would you have liked them to have been involved more?*
- *Can you give me an example? Have they been involved too much?*

**14. Can you suggest anything that would improve care planning, either for you or**

**generally?**

*Prompt:*

- *Anything that could be done differently or a new approach to doing things?*
- *Can you tell me more about that idea? How would that improve things?*

**15. Is there anything else you would like to say that we have not covered?**

*Prompt:*

- *Is there anything we haven’t asked you that we should have?*

**Ok, that’s the end of the interview. Thank you very much for your time.**
