# Supplementary material for: Recovery-focused care planning and coordination in England and Wales: a cross-national mixed methods comparative case study
Source: BMC Psychiatry. 2016 May 16;16:147. doi: 10.1186/s12888-016-0858-x (PMC4868048; doi:10.1186/s12888-016-0858-x)
Supplement: Additional file 2: — Care coordinator semi-structured interview COCAPP, interview schedule. (DOCX 123 kb) [file 12888_2016_858_MOESM2_ESM.docx]

# COCAPP – Collaborative Care Planning Project

**Semi-Structured Interview Schedule**

**Care Coordinator Version 2**

Introduce yourself and explain nature of the study:

**Hi. My name is XXXX. Thank you for meeting with me today.**

**You kindly agreed to take part in the COCAPP research project and I am here today to ask you a few questions about your experience of care planning and coordination. It should take about 45 minutes at most. There are no right or wrong answers. We just want to know what you think about the way care for service users is planned and coordinated.**

Remind the person that they have already given their consent to be interviewed and check that they are still OK with that. Remind them their name will not be used and they will not be identified in any way. They may stop at any time.

Check digital recorder and microphone are working and sound levels are adequate.

**I am just going to read out the code number for you in this study so that your name can be left out of it and the interview remains anonymous.**

*Read out Participant Code and Date.*

1. **What does the CPA/Care and Treatment Planning mean to you?**

*Prompts:*

- *What is the purpose of care planning?*
- *What are the most important aspects of CPA/CTP?*
- *What works/doesn’t work?*
- *Have your views about CPA/CTP changed at all over time? In what way/why?*

1. **Can you tell me how care for a service user is planned by you and the**

**Community Mental Health Team?**

*Prompts:*

- *Perhaps you could give me an example of one of your service users – how do you go about planning care for Mr Y?*
- *How do care plans fit in with this?*
- *How are care plans useful/helpful?*
- *Could we do things without care plans? How?*
- *Would a care plan in different formats be helpful (e.g. as a phone app?)*
- *When and how often do you refer to the care plan?*
- *Who else uses the care plan? When?*
- *To what extent can service users have their say in what is in the care plan?*
- *How did you involve Mr Y in his care planning?*
- *What challenges are there in involving the service user?*
- *How do agree what goes in the care plan?*
- *How do you resolve disagreements?*
- *How do you decide on priorities in a care plan?*
- *Who owns the care plan?*

**3. What happens when the service user’s care is reviewed?**

*Prompts:*

- *Do you have care review meetings? How helpful are they?*
- *What do you find helpful? Less helpful?*
- *Do you have enough time?*
- *Who is involved in those reviews?*
- *Do you have any choice about the timing, venue or who chairs the meeting?*
- *How are you as a care coordinator involved? Are you able to contribute?*
- *How are service users involved? Are their views listened to? Are their wishes and preferences taken on board?*
- *How do you help ensure service users are involved?*
- *What would help service users to be more involved?*

**4. Please tell me about your role as a Care Coordinator.**

*Prompt:*

- *What is the purpose of care coordination?*
- *What does care coordination mean to you?*
- *How do you coordinate care?*
- *What are the most important aspects of the role?*
- *What does a service user get from you as their care coordinator?*
- *What aspects of the role work/what doesn’t work?*
- *What training/preparation for the care coordinator role have you had? When was that?*
- *Can you tell me about the content of the training?*
- *How much involvement in a service user’s care is there from other members of the CMHT? From other agencies/organisations/ groups?*
- *How do you coordinate that input?*

**6. Lots of people talk about Recovery in mental health nowadays – what does the**

**term Recovery mean to you?**

*Prompt:*

*Thank you, that’s helpful. For many people, Recovery is generally seen as a personal journey for the service user ... one that may involve developing hope, a secure base and sense of self, supportive relationships, being more in control of their life and care, social inclusion, and how developing coping skills... often despite still have symptoms of mental illness etc*

*[ask next question]*

**7. How has care planning and coordination been changed or influenced by the**

**Recovery approach?**

*Prompt:*

- *Has it made any difference? In what way?*
- *Do you think you are work in a Recovery-focused way? Can you give me an example?*
- *Are service users encouraged to develop Personal Recovery Plans or Wellness Recovery Action Plans (WRAP)?*
- *How much are you able to focus on the service user’s abilities, assets, skills, strengths? Could you give me some examples?*
- *What difficulties are there with a Recovery-focused approach?*

**10. Another term that is being used a lot is ‘Personalisation’ - what does the term**

**‘Personalisation’ mean to you?**

*Prompt:*

*Thank you, that’s helpful. For many people, Personalisation is often seen as putting service users firmly in charge of their care and support and that care is designed with their full involvement and tailored to meet their own unique needs. [ask next question]*

**11. Do you think your approach to care and treatment planning with service users**

**is personalised?**

*Prompt:*

- *Could you give me an example of where someone’s care was personalised? In what way was it not personalised?*
- *Could you give me an example of how care is personalised/not personalised?*
- *Is care tailored towards a service user’s individual needs?*
- *Do service users feel in charge of their care and support?*
- *What difficulties are there with a Personalised approach?*

**12. How do you consider issues of safety and risk when planning and**

**coordinating care?**

*Prompts: [Acknowledge if person has already mentioned safety/risk – would you like to add anything else?]*

- *How do you considered the safety of the service user?*
- *How about the safety of others?*
- *How does the focus on risk sit alongside other aspects of care planning and coordination?*

**13. Can you tell me how carers, family members/friends are involved in care**

**planning and reviews?**

*Prompt:*

- *Could you give me an example of involving carers/family?*
- *How typical or common is it to involve carers?*
- *What are the challenges for involving carers/family?*
- *Would you like them to be involved more?*
- *Can carers be involved too much?*
- *How things could be improved?*
- *Is there anything that should be happening and isn’t or something happening which shouldn’t be?*

**14. Can you suggest an intervention that would improve care planning?**

*Prompt:*

- *Anything that could be done differently or a new approach to doing things?*
- *Can you tell me more about that idea? How would that improve things?*

**15. Is there anything else you would like to say that we have not covered?**

*Prompt:*

- *Is there anything we haven’t asked you that we should have?*

**Ok, that’s the end of the interview. Thank you very much for your time.**
