# Supplementary material for: Recovery-focused care planning and coordination in England and Wales: a cross-national mixed methods comparative case study
Source: BMC Psychiatry. 2016 May 16;16:147. doi: 10.1186/s12888-016-0858-x (PMC4868048; doi:10.1186/s12888-016-0858-x)
Supplement: Additional file 3: — Carer semi-structured interview COCAPP, interview schedule. (DOCX 125 kb) [file 12888_2016_858_MOESM3_ESM.docx]

# COCAPP – Collaborative Care Planning Project

**Semi-Structured Interview Schedule**

**Carer Version 2: 08.02.2013**

Introduce yourself and explain nature of the study:

**Hi. My name is XXXX. Thank you for meeting with me today.**

**You kindly agreed to take part in the COCAPP research project and I am here today to ask you a few questions about your experience of care planning and coordination. It should take about 45 minutes at most. There are no right or wrong answers. We just want to know what you think about the way the care has been planned and coordinated for [your family member/friend – insert name].**

Remind the person that they have already given their consent to be interviewed and check that they are still OK with that. Remind them their name will not be used and they will not be identified in any way. They may stop at any time.

Check digital recorder and microphone are working and sound levels are adequate.

**I am just going to read out the code number for you in this study so that your name can be left out of it and the interview remains anonymous.**

*Read out Participant Code and Date.*

**1. Can you tell me how [Name’s] care is planned by the Community Mental Health Team?**

*Prompts:*

- *Does he/she have a written care plan?*
- *What do you find helpful? Less helpful?*
- *Did you receive a copy of the care plan?*
- *Was that helpful? Do you understand the care plan? [If no care plan] – How would a copy of the care plan have been helpful to you?*
- *Would a care plan in different formats be helpful (e.g. as a phone app?)*
- *Were you aware of his/her care being planned?*
- *How were you involved in the planning of his/her care?*
- *What would help you to be more involved?*
- *What is important for you?*
- *When and how often do you refer to the care plan?*

**2. Can you tell me about what happens when [Name’s] care is reviewed?**

*Prompts:*

- *Are there care review meetings?*
- *How helpful are they? What do you find helpful? Less helpful?*
- *Do you have enough time?*
- *Who is involved in those reviews? Are you involved in the review meetings?*
- *Do you have any choice about the timing, venue or who chairs the meeting?*
- *How were you involved? Could you contribute? Were your views listened to?*
- *Are your wishes and preferences taken on board?*
- *What would help you to be more involved?*
- *Have you had any experience of meetings with [Name’s]* *care coordinator to prepare for review meetings?*

**3. Please tell me about [Name’s] Care Coordinator**

*Prompt:*

- *What sort of support does [Name] get from him/her?*
- *Does [Name] have a good relationship with him/her?*
- *Do you have a good relationship with him/her?*
- *How long have you known this care coordinator?*
- *Have you known others? Was that similar or different?*
- *What do they do well/less well?*
- *Are you aware of [Name’s] care being coordinated? What does that mean to you?*

**4. What sort of support do you get from the Care Coordinator?**

*Prompt:*

- *Do you meet up? Does s/he phone you at all? Anything else?*
- *What is most helpful? What do they help you with?*
- *Are there things you would like more help with? What sort of things?*
- *How often do you see him/her?*
- *Do you feel able to be open/express your fears with [Name’s] care coordinator?*
- *Do you feel you trust [Name’s] care coordinator?*

**5. Does [Name] have help or support from other workers?**

*Prompt:*

- *Like who? Social worker? Mental Health Nurse? Psychologist? Occupational therapist? Support worker?*
- *What has that been like?*
- *Does there appear to be communication between these different workers?*
- *Is the care coordinator involved in that?*
- *Did they have contact with you? Have you been offered support?*
- *From who? What information have you been given about other forms of support (e.g. carer support groups, carer’s assessment, benefits advice)?*

**6. Would you have liked to have had more of anything either for [Name] or yourself?**

*Prompt:*

*What sort of thing would you have liked the Care Coordinator to have done more?*

**7. Is there anything that you didn’t like about the way [Name’s] care was planned?**

*Prompt:*

*Was there anything that was unhelpful? Intrusive?*

**8. Lots of people talk about Recovery in mental health nowadays – what does the term**

**Recovery mean to you?**

*Prompt:*

*Thank you, that’s helpful. For many people, Recovery is generally seen as a personal journey ... one that may involve developing hope, a secure base and sense of self, supportive relationships, being more in control of one’s life and care, social inclusion, and how to cope... often despite still have symptoms of mental illness etc [ask next question]*

**9. How has the planning of [Name’s] care helped with his/her Recovery?**

*Prompt:*

- *Has [Name] been encouraged to develop a Personal Recovery Plan? A Wellness Recovery Action Plan (WRAP)?*
- *Have there been things that have helped his/her Recovery?*
- *Are there things you think might have helped his/her Recovery?*
- *Are his/her achievements recognised? Are they recorded?*

**10. Another term that is being used a lot is ‘Personalisation’ - what does the term**

**‘Personalisation’ mean to you?**

*Prompt:*

*Thank you, that’s helpful. For many people, Personalisation is often seen as putting service users firmly in charge of their care and support and that care is designed with their full involvement and tailored to meet their own unique needs. [ask next question]*

**11. Do you think [Name’s] care and treatment was personalised?**

*Prompt:*

*Did you think [Name’s] care was tailored towards him/her and his/her individual needs? What does personalised care mean to you? Could you give me an example of where you think [Name’s] care was personalised? In what way was it not personalised? How much influence does [Name] have over his/her care and support provided?*

**12. Do you feel your safety and/or the safety of [Name] has been considered in their care planning and coordination?**

*Prompts:*

- *How has the safety of [Name] been addressed in his/her care plan or by the care coordinator?*
- *How has your safety been addressed?*
- *Have any other aspects of safety or risk been discussed with you?*

**13. Can you suggest anything that would improve care planning for [Name] or generally?**

*Prompt:*

- *Anything that could be done differently or a new approach to doing things?*
- *Can you tell me more about that idea? How would that improve things?*

**14. Is there anything else you would like to say that we have not covered?**

*Prompt:*

- *Is there anything we haven’t asked you that we should have?*

**Ok, that’s the end of the interview. Thank you very much for your time.**
