# Supplementary material for: Recovery-focused care planning and coordination in England and Wales: a cross-national mixed methods comparative case study
Source: BMC Psychiatry. 2016 May 16;16:147. doi: 10.1186/s12888-016-0858-x (PMC4868048; doi:10.1186/s12888-016-0858-x)
Supplement: Additional file 4: — Managers semi-structured interview COCAPP, interview schedule. (DOCX 120 kb) [file 12888_2016_858_MOESM4_ESM.docx]

# COCAPP – Collaborative Care Planning Project

**Semi-Structured Interview Schedule**

**Manager/Senior Practitioner Version 2: 11.02.2013**

Introduce yourself and explain nature of the study:

**Hi. My name is XXXX. Thank you for meeting with me today.**

**You kindly agreed to take part in the COCAPP research project and I am here today to ask you a few questions about the recent history and development of care planning and coordination and the impact of Recovery and personalisation.**

**It should take about 45 minutes at most. There are no right or wrong answers. We just want to know what you think about the way care for service users is planned and coordinated.**

Remind the person that they have already given their consent to be interviewed and check that they are still OK with that. Remind them their name will not be used and they will not be identified in any way. They may stop at any time.

Check digital recorder and microphone are working and sound levels are adequate.

**I am just going to read out the code number for you in this study so that your name can be left out of it and the interview remains anonymous.**

*Read out Participant Code and Date.*

1. **What does the CPA/Care and Treatment Planning and Coordination mean to you?**

*Prompts:*

- *What is the purpose of care planning and coordination?*
- *What are the most important aspects of CPA/CTP?*
- *What works/doesn’t work?*
- *Who receives care/support under CPA/CTP? Who is not included?*
- *Who can take on the Care Coordinator role?*
- *Do some disciplines have different roles within the care planning/coordination process?*
- *What training do care coordinators receive?*
- *What are the major challenges faced by your Trust/Board in relation to care planning and coordination?*
- *What helps/hinders CPA/CTP?*

1. **What sort of care planning documentation do you use?**

*Prompts:*

- *Has that changed at all in the last 3-5 years?*
- *How has the documentation changed/developed? [seeking evidence of changes to reflect Recovery and personalisation without being explicit]*
- *Could we obtain a copy of any documents used please?*
- *How are care reviews organised?*
- *How often do they take place?*

1. ***Do you know how many patients are on CPA/CTP per CMHT and per care coordinator?***

*Prompts:*

- *Have those numbers changed over the last three years?*
- *How many people in your Trust/Health Board and as a proportion of the population you serve?*

1. **What have been the key developments in community mental health services here over the last 3-5 years?**

*Prompts:*

- *Who and what is driving local development of community mental health*

*services?*

- *Who are the key people?*
- *What are the key policies?*
- *Has there been any funding for specific projects/developments?*
- *How has that impacted on care planning and coordination?*
- *How about Payment by Results (PbR) and Clustering (England only)? Other specific policies?*
- *Has that impacted on care planning and coordination?*

1. **Have there been any significant changes/developments in relations between health and social services?**

*Prompts: Has that impacted on care planning and coordination? In what way?*

1. **What are the values or principles that underpin the provision of CMH services in this Trust/Health Board?**

*Prompts:*

- *What are the relationships between these values/principles and care planning and coordination?*

1. **There is an increasing focus on Recovery in mental health nowadays – what**

**does the term Recovery mean to you?**

*Prompt:*

*Thank you, that’s helpful. For many people, Recovery is generally seen as a personal journey for the service user ... one that may involve developing hope, a secure base and sense of self, supportive relationships, being more in control of their life and care, social inclusion, and how developing coping skills... often despite still have symptoms of mental illness etc [ask next question]*

**8. How has care planning and coordination been changed or influenced by the**

**Recovery approach in your Trust/Health Board?**

*Prompt:*

- *Has it made any difference? In what way?*
- *Do you think your services are Recovery-focused? Can you give me an example?*
- *Are service users encouraged to develop Personal Recovery Plans or Wellness Recovery Action Plans (WRAP)?*
- *How much are you able to focus on the service user’s abilities, assets, skills, strengths? Could you give me some examples?*
- *What difficulties or challenges are there with a Recovery-focused approach?*
- *Does the Trust/Health Board have a Recovery policy? Could we obtain a copy of that please?*

**9. Another term that is being used a lot is ‘Personalisation’ - what does the term**

**‘Personalisation’ mean to you?**

*Prompt:*

*Thank you, that’s helpful. For many people, Personalisation is often seen as putting service users firmly in charge of their care and support and that care is designed with their full involvement and tailored to meet their own unique needs. [ask next question]*

**10. Do you think your Trust/Health Board’s approach to care and treatment**

**planning with service users is personalised?**

*Prompt:*

- *What changes have been made to make care planning and coordination more personalised? Could you give me an example?*
- *Could you give me an example of how care is personalised/not personalised?*
- *Is care tailored towards a service user’s individual needs?*
- *How much are Personal Budgets used here?*
- *Do service users feel in charge of their care and support?*
- *What difficulties or challenges are there for you to deliver a Personalised approach?*
- *Does the Trust/Health Board have a Recovery policy? Could we obtain a copy of that please?*

**11. How are issues of safety and risk considered when planning and coordinating**

**care?**

*Prompts:*

*[Acknowledge if person has already mentioned safety/risk – would you like to add anything else?]*

- *How do you consider the safety of the service user?*
- *How about the safety of others? How does the focus on risk sit alongside other aspects of care planning and coordination?*

**12. Can you tell me how carers, family members/friends are involved in care**

**planning and reviews?**

*Prompt:*

- *How good do you think your Trust/Health Board are at ensuring Families/Carers are involved and supported?*
- *Does your Trust/Health Board have a policy on involvement of families and carers? How typical or common is it to involve carers?*
- *What are the challenges for involving carers/family?*
- *Would you like them to be involved more?*
- *Can carers be involved too much? How things could be improved?*

**13. Can you suggest anything that would improve care planning and coordination**

**in your Trust/Health Board?**

*Prompt:*

- *Is there anything that could be done differently or a new approach to doing things?*
- *Can you tell me more about that idea?*
- *How would that improve things?*

**14. What are the three key factors that would help you ensure that your services**

**deliver recovery-focused and personalised care and treatment?**

*Prompt: What would help you as a manager/senior practitioner?*

**15. What are the three key challenges you face in ensuring that your services deliver recovery-focused and personalised care and treatment?**

*Prompt: What are the biggest challenges for you as a manager/senior*

*practitioner?*

**16. Is there anything else you would like to say that we have not covered?**

*Prompt: Is there anything we haven’t asked you that we should have?*

**Ok, that’s the end of the interview. Thank you very much for your time.**
